# The efficacy and safety of acupuncture or combined with western medicine for dry eye: A protocol for systematic review and meta-analysis

**DOI:** 10.1097/MD.0000000000021878

**Published:** 2020-08-28

**Authors:** Xu Wang, Yihua Fan, Hongyi Lan, Yanjin Song, Zongyan Shi, Zhillong Zhang, Xinju Li

**Affiliations:** a Tianjin Academy of Traditional Chinese Medicine Affiliated Hospital; b First Teaching Hospital of Tianjin University of Traditional Chinese Medicine; c Tianjin University of Traditional Chinese Medicine, Tianjin, China.

**Keywords:** acupuncture, dry eye, meta-analysis, protocol, systematic review

## Abstract

**Background:**

As a traditional Chinese medicine external treatment method, acupuncture is characterized by simple operation, significant treatment effect and few side effects. Dry eye, also known as conjunctival xerosis, refers to pathological changes in tissues of ocular surface caused by abnormal tear quality and quantity or dynamics due to various reasons, which can cause red and swollen eyes, congestion, and severe cases may result in lesions of the conjunctiva. Clinical practice shows that acupuncture has a certain therapeutic effect on dry eye, but there is a lack of medical evidence. The objective of the research carried out in this program is to evaluate the effectiveness and safety of acupuncture combined with western medicine in the treatment of dry eye.

**Methods:**

Computer retrieval English database (PubMed, Embase, Web of Science, the Cochrane Library) and Chinese database (CNKI, WF, VIP, CBMDISC), moreover manual retrieval of clinical research on the treatment of dry eyes by acupuncture in combination with western medicine from Baidu academic and Google Academic from database building to May 2020, the quality included in the study was evaluated and data extraction was completed by 2 independent researchers. RevMan5.3 software was utilized for meta-analysis to the included literature.

**Results:**

In this study, the effectiveness and safety of acupuncture combined with western medicine in the treatment of dry eye were evaluated by several indicators, such as the score of the ocular surface disease index, the amount of tear secretion and the tear film rupture time.

**Conclusions:**

This study will provide reliable evidence for the clinical application of acupuncture combined with western medicine in the treatment of dry eyes.

**OSF Registration number:**

DOI 10.17605/OSF.IO/5DJ9W.

## 1 Introduction

Dry eye is a dry eye phenomenon that occurs mainly due to the pathology of the conjunctival tissue. In recent years, dry eye has been defined as a multifactorial disease characterized by abnormal homeostasis of tear film and ocular surface symptoms. Common symptoms include dry eyes, fatigue, itchy eyes, foreign body sensation, burning sensation, thick discharge, fear of wind, photophobia, etc. The causes include tear film instability, tear hypertonicity, inflammation and damage on ocular surface, and neurosensory disorder.^[1]^ Chinese scholars believe that tear film instability is the core mechanism of dry eye, while increased tear permeability and ocular surface inflammation are a series of secondary pathophysiological changes that occur after tear film instability.^[2]^ At present, western medicine treats the disease with artificial tears, artificial solutions, immunosuppression, glucocorticoids, surgery method, etc.^[3]^ Although it can temporarily improve symptoms, adverse reactions and complications of different degrees occur.

As one of the characteristic therapies of traditional Chinese medicine, acupuncture can regulate the movement of channel qi and improve the nourishment of blood in the eyes.^[4]^ Clinical studies have found that acupuncture can promote tear secretion and improve dry eyes.^[5]^ In addition, acupuncture is easy to operate with safe, effective treatment and small adverse reactions, without any toxic effects. Therefore, it has certain advantages in clinical practice.^[6]^

At present there are multiple randomized controlled study results show that, acupuncture has a significant effect on dry eye with high cure rate, low recurrence rate and less adverse reaction, but there exist great differences in the design and efficacy of clinical study, leading to the uneven results. To a certain extent, that affects the reliability of the study results and the promotion of the therapy. Therefore, the objective of the study is to evaluate the efficacy and safety of acupuncture alone or combined with western medicine in the treatment of dry eye, so as to provide a reliable basis for the clinical treatment of dry eye with acupuncture.

## 2 Methods

### 2.1 Protocol register

This protocol of systematic review and meta-analysis has been drafted under the guidance of the preferred reporting items for systematic reviews and meta-analyses protocols (PRISMA-P). Moreover, it has been registered on open science framework (OSF) on July 21, 2020 (Registration number: DOI 10.17605/OSF.IO/5DJ9W).

### 2.2 Ethics

Since this is a protocol with no patient recruitment and personal information collection, the approval of the ethics committee is not required.

### 2.3 Eligibility criteria

#### 2.3.1 Types of studies

We will collect all available randomized controlled trails on acupuncture treatment for dry eye, regardless of blinding, publication status, region, but Language will be restricted to Chinese and English.

#### 2.3.2 Objects of the study

Patients definitely diagnosed with dry eye but without other complications, including their nationality, race, age, sex, course of disease and pathogenic site

#### 2.3.3 Intervention

The treatment group was treated by filiform needle acupuncture combined with western medicine, including acupuncture and acupuncture combined with western medicine. There were no restrictions on filiform needle acupuncture techniques and acupoints; the control group was treated by western medicine alone where type, dose and course of treatment were not defined.

#### 2.3.4 Outcome indicator

(1) Primary outcome: Ocular surface disease index;
(2) Secondary outcomes: Schirmer test, Tear break-up time, lactoferrin in tears, Visual analogue scale, Incidence of adverse reactions.

### 2.4 Exclusion criteria

(1) Study published repeatedly;
(2) Study whose literature is abstract and conference papers, in which the original data cannot be obtained;
(3) Study whose data is incomplete or where there are obvious errors that cannot be handled after contacting the author;
(4) Study with wrong random method;
(5) Study where the filiform acupuncture therapies are not used in the treatment group, such as ear acupuncture, moxibustion and acupoint application;
(6) The control group in which the treatment is done by traditional Chinese and western medicine.

### 2.5 Search strategy

The “acupuncture,” “needling,” and “dry eye” were used as Chinese terms to search in Chinese databases, including China Knowledge Network (CNKI), Wanfang Data Knowledge Service Platform, VIP Information Chinese Journal Service Platform (VIP), China Biomedical Database; “acupuncture,” “needling,” “dry eye,” used as English terms to search in English databases, including PubMed, EMBASE, Web of Science, the Cochrane Library, and manually search in Baidu academics and Google academics. The retrieval time was from the establishment of the database to July 2020, before all domestic and foreign literatures on the treatment of dry eye with filiform needle acupuncture were collected. Taking PubMed as an example, the search strategy is shown in Table 1.

**Table 1:**
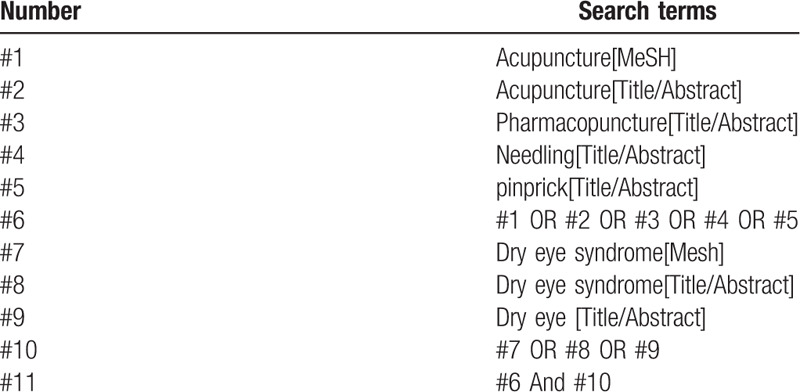
Search strategy in PubMed database.

### 2.6 Data screening and extraction

**Figure 1:**
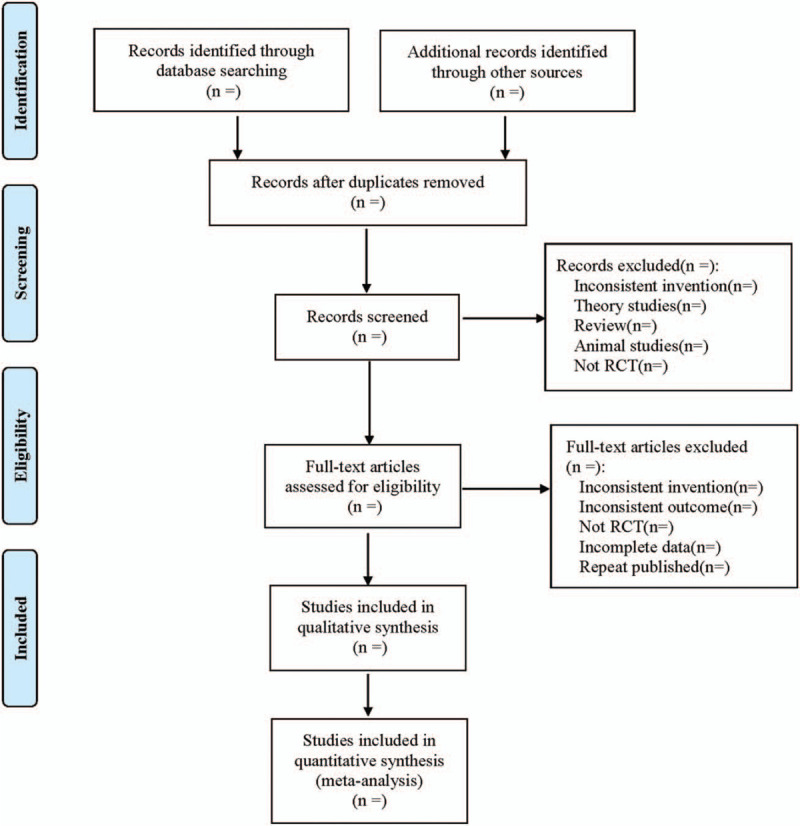
Flow diagram.

Cochrane Collaboration System Reviewer Manual Version 5.0 was used as a reference for the method of selection in the study. According to the PRISMA flow chart, EndNote X7 document management software was utilized by 2 researchers to independently screen the documents based on the above inclusion and exclusion criteria before mutual check. Those difficult to determine whether included in the study, would be discussed and judged with a third researcher. At the same time, Excel 2013 was used to extract relevant information, including:

1. Clinical research (title, first author, publication year and month, sample size, sex ratio, average age, average course of disease);
2. Intervention measures (name of western medicine used in the control group, dosage, course of treatment; acupuncture points, frequency of acupuncture and course of treatment used in the treatment group);
3. Evaluation factors of risk bias in randomized controlled studies;
4. Observation indicators. The literature screening process is shown in Figure 1.

### 2.7 Literature quality evaluation

Built-in Risk bias evaluation tool of Review Manager 5.3 Software (the Cochrane collaboration's tool for assessing risk of bias) was used to assess the risk bias in the included studies. Two researchers determined the literatures from 3 levels, including low-risk, unclear, and high-risk based on the performance of the included literature in the above evaluation items. After completion, they would recheck. In case of a disagreement, they would discuss. If no agreement could be reached, a decision would be made in consultation with researchers from the third party.

### 2.8 Statistical analysis

#### 2.8.1 Data analysis and processing

The RevMan 5.3 software provided by the Cochrane Collaboration was used for statistical analysis.

1. For dichotomous variables, relative risk was used for statistics. For continuous variables, Weighted Mean Difference was selected when the tools and units of measurement indicators are the same, Standardized Mean Difference was selected with different tools or units of measurement, and all the above were represented by effect value and 95% confidence interval (CI).
2. Heterogeneity test: *Q* test was used to qualitatively determine inter-study heterogeneity. If *P *≥* *.1, there was no inter-study heterogeneity, If *P *<* *.1, it indicated inter-study heterogeneity.

At the same time, *I*^2^ value was used to quantitatively evaluate the inter-study heterogeneity. If *I*^*2*^* *≤* *50%, the heterogeneity was considered to be good, and the fixed-effect model was adopted. If *I*^*2*^* *>* *50%, it was considered to have significant heterogeneity, the source of heterogeneity would be explored through subgroup analysis or sensitivity analysis. If there was no obvious clinical or methodological heterogeneity, it would be considered as statistical heterogeneity, and the random-effect model would be used for analysis. Descriptive analysis was used if there was significant clinical heterogeneity between the 2 groups and subgroup analysis was not available.

#### 2.8.2 Dealing with missing data

If data is missing or incomplete, we will contact the corresponding author to obtain the missing data. If not, this study will be removed.

#### 2.8.3 Subgroup analysis

According to the treatment group, acupuncture combined with western medicine and western medicine alone were used for subgroup analysis; according to the age of patients, they could be divided into minors, young people and the elderly for subgroup analysis; according to the acupuncture, the treatment method and the course of acupuncture could be used for subgroup analysis.

#### 2.8.4 Sensitivity analysis

In order to test the stability of meta-analysis results of outcomes, a one-by-one elimination method will be adopted for sensitivity analysis.

#### 2.8.5 Assessment of reporting biases

For the major outcome indicators, if the included study was ≥10, funnel plot was used to qualitatively detect publication bias. Egger and Begg test are used to quantitatively assess potential publication bias.

#### 2.8.6 Evidence quality evaluation

The Grading of Recommendations Assessment, Development, and Evaluation (GRADE)^[23]^ will be used to assess the quality of evidence. It contains 5 domains (bias risk, consistency, directness, precision, and publication bias). And the quality of evidence will be rated as high, moderate, low, and very low.

## 3 Discussion

From the perspective of traditional Chinese medicine, Dry Eye belongs to the categories of “ocular dryness” (BaiSeZheng), “dried-up holy water” (ShenShuiJiangKu), and “dryness syndrome” (ZaoZheng). *General Treatise on the Cause and Symptoms of Diseases (Zhubing Yuanhou Lun)* believes that the disease is caused by emotionally internal injuries, external attacks of wind and pathogens, making the viscera and body fluid deficient, unable to rise to the eyes and ultimately leading to the occurrence of dry eyes.^[7]^ Traditional Chinese medicine clinical syndrome differentiation is generally divided into: dampness and heat and Yin deficiency, liver depression and stagnation, Yin deficiency of lungs, deficiency of both Qi and Yin, deficiency of liver and kidney.^[8]^ The liver is related to the eyes and contains the blood, while the kidneys dominate water and the eyes can be nourished. If the liver and kidney are inadequate, the Yin and Qi are lacking, and the eyes become dry and uncomfortable. The critical treatment of Dry Eye is to tonify the liver and kindly, which can regulate the secretion of tears from an overall perspective and fundamentally treat Dry Eye.^[9]^ According to human meridian circulation, stomach meridian of foot-yangming, heart meridian of hand-shaoyin, taiyang small intestine meridian of hand, the urinary bladder meridian of foot-taiyang, allbladder channel of foot-shaoyang, liver channel of foot jueyin, three-jiao channel of hand shaoyang are all related to eyes, and these meridians are mostly used as treatment points.^[10]^ The commonly used acupoints are DU20 (Baihui), EX-HN5 (Taiyang), BL2 (Cuanzhu), BL1 (Jingming), GB1 (Tongzi Liao), ST1 (Chengqi), ST2 (Sibai), LI20 (Yingxiang), LT4 (Hegu), SP6 (Sanyinjiao), ST36 (Zusanli) and so on. The DU20 (Baihui) will be through the governor and conception vessels, smoothing vital essence. The EX-HN5 (Taiyang), BL2 (Cuanzhu), BL1 (Jingming), GB1 (Tongzi Liao) are local acupoints, which can improve ocular symptoms. ST1 (Chengqi), ST2 (Sibai) can regulate the Qi and blood around the eyes; LI20 (Yingxiang) is the intersection point of the hand and foot Yangming meridian, which can adjust the 2 meridians and dredge wind and heat. LT4 (Hegu) is the original point of large intestine meridian of Hand Yangming, which can regulate qi and activate blood circulation. Distal acupoint selection: SP6 (Sanyinjiao), GB37 (Guangming), ST36 (Zusanli), etc.^[11]^ SP6 (Sanyinjiao) can nourish liver and kidney, harmonize Qi and blood. GB37 (Guangming) can smooth obstructions in liver and gallbladder meridians, harmonize Qi and blood, control the good treatment of various eye diseases. ST36 (Zusanli) is a stomach cooperation point, which plays a role in strengthening the body.^[12,13]^

Clinical studies have shown that acupuncture can affect the expression of lactoferrin in tears and mucoprotein, induce the quantitative changes in some proteins of tears,^[14]^ effectively inhibit the inflammatory response of dry eyes, prolong rupture time of tear film, and enhance the stability of tear film.^[15,16]^ Acupuncture can affect the tissue morphology of functional units, restore damaged corneal tissue, improve dry corneal morphology, and promote the rehabilitation of ocular surface structures,^[17]^ activate the synthesis function of lacrimal gland, restore secretion function, and improve the inherent morphology of the lacrimal gland^[18]^; Acupuncture can affect the nerve regulation of tear secretion, effectively stimulate the activity of parasympathetic nerves,^[19]^ promote the release of acetylcholine from nerve endings, and increase the content of acetylcholine in the lacrimal glands, thereby increasing the secretion of tears^[20]^; It can reduce the inflammation of the ocular surface, effectively reduce the concentration of tumor necrosis factor and interleukin 4 in the tears of patients, and improve the signs of patients with Dry Eye^[21]^; Acupuncture can inhibit cytotoxicity and alleviate cell apoptosis in lacrimal glands and ocular surface epithelial tissues in patients with dry eye^[22,23]^; acupuncture can adjust the level of hormones and regulate the related functions of ocular surface hormone receptors.^[24]^ Acupuncture can regulate the blood circulation around the eyes, promote self-repair of eye tissues, improve ocular symptoms,^[25]^ adjust the quality and quantity of tears, and improve the ocular microenvironment.^[26]^ It is worth noting that during the treatment of Dry Eye by acupuncture, operators shall pay attention to the depth and technique of acupuncture when needling on the points, such as Jingming and Chengqi. Acupuncture should not be too deep or violent to avoid puncturing blood vessels and causing hematoma.

At present, acupuncture therapy alone or combined western medicine for the treatment of dry eye in randomized controlled study was widely reported, but lacking in systematic and correct evaluation. Therefore, it is necessary to objectively evaluate the clinical effectiveness and safety of acupuncture in the treatment of Dry Eye through evidence-based medicine, so as to provide a reliable basis for clinical doctors to take acupuncture in the treatment of Dry Eye and to reduce the adverse reactions of western medicine treatment. However, this study also has some limitations, since the simulate operation is difficult to achieve, it is difficult to implement the blind method. The different acupoints of acupuncture have certain influence on the study results. At the same time, only English and Chinese literature were searched due to the limitation on languages. Studies in other languages were ignored, which might lead to certain publication bias.

## Author contributions

**Data collection:** Hongyi Lan and Yanjin Song.

**Funding to support:** Zhillong Zhang and Xinju Li.

**Literature search:** Xu Wang and Yihua Fan.

**Software operation:** Zongyan Shi.

**Supervision:** Zhillong Zhang and Xinju Li.

**Writing – original draft:** Xu Wang and Yihua Fan.

**Writing – review & editing:** Xu Wang and Zhillong Zhang.
